# Nuclear energy: Twitter data mining for social listening analysis

**DOI:** 10.1007/s13278-023-01033-8

**Published:** 2023-02-06

**Authors:** Enara Zarrabeitia-Bilbao, Maite Jaca-Madariaga, Rosa María Rio-Belver, Izaskun Álvarez-Meaza

**Affiliations:** 1 Faculty of Engineering, University of the Basque Country (UPV/EHU, Bilbao, Spain; 2 Faculty of Engineering, University of the Basque Country (UPV/EHU, Vitoria-Gasteiz, Spain

**Keywords:** Nuclear energy, Twitter, Social network analysis, Artificial neural networks, Russia–Ukraine conflict

## Abstract

Knowing the presence, attitude and sentiment of society is important to promote policies and actions that influence the development of different energy sources and even more so in the case of an energy source such as nuclear, which has not been without controversy in recent years. The purpose of this paper was to conduct a social listening analysis of nuclear energy using Twitter data mining. A total of 3,709,417 global tweets were analyzed through the interactions and emotions of Twitter users throughout a crucial year: 6 months before and 6 months after the beginning of Russian invasion of Ukraine and the first attack on the Zaporizhzhia NPP. The research uses a novel approach to combine social network analysis methods with the application of artificial neural network models. The results reveal the digital conversation is influenced by the Russian invasion of Ukraine. However, tweets containing personal opinions of influential people also manage to enter the digital conversation, defining the magnitude and direction of the debate. The digital conversation is not constructed as a public argument. Generally, it is a conversation with non-polarized communities (politics, business, science and media); neither armed conflict or military threats against Zaporizhzhia NPP succeed in rousing anti-nuclear voices, even though these events do modify the orientation of the sentiment in the language used, making it more negative.

## Introduction

It is an undeniable fact that in recent years there has been an intense worldwide public debate on nuclear energy (Diaz-Maurin 2014; Diaz-Maurin and Kovacic 2015). The urgency of mitigating climate change, as well as dependence on fossil fuels, has reopened the debate on this energy source (Friederich and Boudry 2022). Thus, while its detractors call for its abolition due to its dangerousness and the problems derived from waste generation (Greenpeace 2022; NRDC 2022), its defenders fundamentally see it as a way toward decarbonization (IAEA 2020; NEI 2022).

The United States (historical supporter of this energy source and country with most nuclear power plants in operation (IAEA-PRIS 2022) is cautiously embracing nuclear power, despite certain environmentalists’ persistent concerns, to help achieve its goal of a net-zero carbon economy for the nation by 2050 (USDS-USEOP 2021). Japan (country operating the third most nuclear power plants), where nuclear power has been anathema since the Fukushima Daiichi nuclear power plant disaster in 2011 (Schneider and Froggatt 2012), is advocating the operation of reactors that have remained inactive for ten years since the accident (Reuters 2021).

There is no doubt that nuclear energy-related developments have also been compulsive in Europe. In November 2021, France (country operating the second most nuclear power plants) announced that it will relaunch the construction of nuclear reactors in the country (LeFigaro 2021), thus reversing the discussion on an energy source that seemed obsolete after the catastrophe at the Fukushima plant. In addition, on New Year’s Eve 2021, nuclear power experienced a partial shutdown in Berlin and a resurgence in Brussels: Germany shut down three of its six operating nuclear power plants as part of the approved plan to shut down all atomic production by the end of 2022 (Joly 2021). However, at the same stroke of midnight, the European Commission announced its pioneering proposal to change the classification of green energy to include nuclear power and natural gas as green energy (European Commission 2022).

In this scenario, however, an event that has changed the worldwide debate about nuclear energy has been the Russian invasion of Ukraine on February 24, 2022. The armed conflict between Russia and Ukraine has undoubtedly influenced energy policies around the world and has led countries to push for a rapid transition to greener energy sources. Nuclear power has been in decline since the Fukushima disaster more than a decade ago, but the energy and environmental crisis is prompting reappraisal of the industry. Hence, the Russian invasion of Ukraine; rising natural gas prices; and Russian energy dependence have reopened the debate on whether nuclear power can help solve the challenges of energy security and climate change.

Nonetheless, the March 4, 2022, attack of the Zaporizhzhia nuclear power plant (NPP) in Ukraine (the largest nuclear power plant in Europe) has caused enormous social upheaval. The attack on the Zaporizhzhia NPP is the first attack, with pernicious intent, on a nuclear power plant (without a radiation accident). There have been previous nuclear disasters such as Kyshtym (USSR, 1957), Windscale Piles (UK, 1957), Three Mile Island (USA, 1979), Chernobyl (USSR, 1986) and Fukushima (Japan, 2011); however, none were intentionally provoked.

Knowing the presence, attitude and sentiment of society is important to promote policies and actions that influence the development of different energy sources (Ibar-Alonso et al. 2022). Hence, being aware that in the post-Fukushima era, nuclear energy became a delicate situation and public opinion became pessimistic (Kim et al. 2016), so analyzing how recent events have affected society can be considered important and of great interest to the scientific community. That is to say, what the social response on this occasion has been and whether it has led to any change in strategy or positioning with respect to this energy source.

To this end, the interactions and discussions generated on Twitter will be analyzed. Twitter is considered a social network that represents an ideal scenario for diverse interactive audiences and a contemporary digital town square (Fernández-Gómez et al. 2018; Orbegozo-Terradillos et al. 2022).

Based on this scenario, the purpose of this paper was to conduct a social listening analysis of nuclear energy. With this purpose, on the one hand, the relationships established (discussion communities generated) on the social network in relation to nuclear energy will be analyzed throughout a whole year and; on the other hand, the overall feelings and attitudes toward nuclear energy will be determined through sentiment analysis of tweets. All this, with special emphasis on the Russian invasion of Ukraine and the Zaporizhzhia NPP attack.

## Twitter data mining and nuclear energy

The information age or information society is defined by the social changes that have taken place since the final decades of the twentieth century, derived from the development of Information and Communication Technologies together with the development of networked social structures, which have impacted all areas of human activity (Mansell 2009; Castells 2010; Del-Fresno-García 2014). Today, a dense social network of interactions links people, information, events, places, etc., facilitating or limiting the flows of information, ideas and perceptions, among others, in an instantaneous and massive networked communication systems (Del-Fresno-García 2014).

In this context, Twitter is a discursive space that has emerged in the heat of the digital agora, allowing people to make themselves visible: being seen and heard under the protection of the most diverse causes, slogans or situations of conformity or nonconformity (Baer 2016). Several studies have shown how Twitter plays a key role in times of crisis and conflict (Orbegozo-Terradillos et al. 2019). In addition, it makes it possible to capture a wide variety of data in real time, and retrospectively, providing access to data records of human activity in the digital sphere over time (Del-Fresno-García 2014).

Nonetheless, the value of the data alone is not high; however, data are the raw material from which more value can be generated. The specific process used to harness data as a raw material and generate is known as data mining, and the operation essentially consists of capturing a series of information records and interpreting them (Morales-i-Gras 2020a). In this regard, numerous researchers have used Twitter data to achieve different objectives related to nuclear energy through the analysis of such data and using various data mining techniques.

In this scenario, it is worth noting that most of the scientific works related to Twitter data mining and nuclear energy refer to the disaster at the Fukushima nuclear power plant. As a consequence of the Fukushima nuclear catastrophe, large amounts of radioactive materials leaked out, causing radioactive pollution of water. Hence, shortly after the catastrophe, public opinion was formed through various platforms, including digital social network services such as Twitter (Seung-Hoi et al. 2016).

The exchange of information on digital social networks has far-reaching positive effects, such as real time and high broadcastability properties. Moreover, Twitter users are simultaneously consumers and contributors of information (Veil et al. 2011). However, in the Fukushima power plant disaster, the information that spread rapidly included misleading reports, such as the claim that iodine is useful for treating radioactivity as a substitute for stable iodine, and that became a problem (Aoki et al. 2018). Risk information transmitted by digital social networks had a strong influence on both the perception of risk and public reactions to an unusual catastrophe (Chung 2018). All in all, after the Fukushima nuclear power plant accident, risk and environmental communication has become a widely studied topic through the social network Twitter (Li et al. 2016). In the months following the accident, science-based tweets decreased; however, tweets with more emotional expressions began to spread and Twitter worked as a new public sphere especially in terms of anti-nuclear movements (Kim 2014). Likewise, it was observed that the role of influencers was crucial for spreading information (Tsubokura et al. 2018; Sano et al. 2021).

In this context, with the emergence of social networks, new ways of expressing opinions have emerged, making the analysis of emotions and feelings displayed on digital platforms key toward better understanding users' opinions on a given topic (Arumugam et al. 2021). Hence, Twitter users’ emotional response after the Fukushima nuclear disaster, mainly negative and opposed to nuclear energy, has been studied in detail (Miura et al. 2015; Su et al. 2016; Kim et al. 2016; Hasegawa et al. 2020). Identifying the process by which people emotionally respond could be useful in risk communication when similar disasters occur in future (Hasegawa et al. 2020).

However, despite the fact that the Fukushima incident has been widely analyzed through Twitter data mining, other fields of study as well as other geographical locations have also been covered, such as political debates about nuclear withdrawal initiatives or narrative policy frameworks (Arlt et al. 2018; Gupta et al. 2018).

In all these studies, various data mining tools, techniques and methodologies are used. One of the techniques widely used is social network analysis (SNA) (Rantasila et al. 2018; Yagahara et al. 2018), which allows knowledge to be generated according to the relational structure of virtual interactions (Morales-i-Gras 2020b). In addition, increasingly sophisticated tools are being used to provide more precise answers to more complex realities. In this sense, within deep learning algorithms, artificial neural networks (ANNs) are used, especially for deciphering public opinion or analyzing emotions and sentiments toward nuclear energy (Liu and Na 2018; Khatua et al. 2020; Arumugam et al. 2021). However, these techniques are usually used separately, i.e., SNA techniques are not combined with ANN.

## Research questions and methodology

The study focuses on the interactions and emotions of Twitter users regarding nuclear energy throughout a crucial year: 6 months before and 6 months after the beginning of Russian invasion of Ukraine and the first attack on the Zaporizhzhia NPP, i.e., from September 1, 2021, to August 31, 2022. Three specific periods have been studied: First period, before the beginning of the conflict; second period, the first three weeks of the conflict, when the first attack on the nuclear power plant took place; and third period, after the beginning of the conflict, i.e., after the first three weeks have elapsed.

For that purpose, the research uses a novel approach to combine social network analysis (SNA) methods with the application of artificial neural network (ANN) models. In other words, a set of methods for the analysis of social interactions that specifically investigate relational structures and their representation as networks (SNA) are combined with algorithms modeled as elementary units or neurons connected in such a way to form a network capable of solving complex nonlinear problems (ANN) (see Analysis methods section).

### Research questions

In order to address the objective of social listening analysis on Twitter about nuclear energy using Twitter data mining, the following questions have been used as a guide:

- *RQ-1*: What have been the main characteristics of the overall conversation about nuclear energy that has taken place throughout a year? Are there morphological differences between the conversations generated before, during and after the beginning of the Russian invasion of Ukraine and the attack on the Zaporizhzhia NPP?
- *RQ-2*: Which have been the main communities generated in the overall conversation about nuclear energy throughout a year? How have these communities changed before, during and after the beginning of the Russian invasion of Ukraine and the attack on the Zaporizhzhia NPP?
- *RQ-3*: Who have been the leaders in the overall conversation about nuclear energy throughout a year? How have these influential players changed before, during and after the beginning of the Russian invasion of Ukraine and the attack on the Zaporizhzhia NPP?
- *RQ-4*: What has been the sentiment about nuclear power throughout a year? How has the sentiment expressed in nuclear energy-related tweets changed before, during and after the beginning of the Russian invasion of Ukraine and the attack on the Zaporizhzhia NPP?

### Research methodology

#### Collecting text data for analysis

The first step, in order to address the main objective of the study, was data extraction for the Twitter social network and data preparation.

In this regard, first, the search query considered to select the tweets that make up the sample of this study was: “nuclear energy” OR nuclearenergy OR “nuclear power” OR nuclearpower. Subsequently, the tweets (from September 1, 2021, to August 31, 2022) were downloaded using the Twitter API for Academic Research (Twitter 2022) and Twarc (a command line tool and Python library for collecting and archiving Twitter JSON data via the Twitter API (Twarc 2022)). The data were collected retroactively in two batches. On August 25, 2022, tweets sent from September 1, 2021, through July 31, 2022, were downloaded, and on September 1, tweets sent throughout August 2022 were downloaded.

Once the tweets were obtained, data preprocessing was necessary for subsequent data analysis. Thus, OpenRefine open-source software was used for data cleanup and transformation (OpenRefine 2022). This cleaning and transformation consisted, mainly, in extracting mentions in order to synthesize different networks based on which users mention which other users in the conversation itself.

#### Analysis methods

The second step was the empirical approach of the research, developed in two stages. In the first stage, networks metrics and main communities were analyzed (answering RQ-1, RQ-2 and RQ-3), and in the second stage, the emotional stage of the digital discussions was studied (answering RQ-4).

To obtain the different discussion networks generated about nuclear energy (a network that takes into account the discussion for the whole year, another network for the first period, another for the second period and the last one for the third period), all the mentions in the digital conversations were extracted, synthesizing networks based on which users mentioned other users in the conversation itself. The resulting networks were exported to the social network analysis open-source software Pajek (Batagelj and Mrvar 2002), and in order to analyze the established relationships between users of the digital conversation sphere, different metrics were obtained, at both the global and individual level (at network level and at nodal level). Hence, through the global metrics, the morphology of the networks was analyzed, and through the metrics at the nodal level, the key actors of the conversations were identified.

The Louvain multi-level algorithm was applied to identify the communities that have participated in the digital conversations. This algorithm allows densely interconnected groups of nodes (Twitter user) to be generated, as well as obtaining the best network partitions or the highest modularity figure. Thus, the groups or clusters representative of the digital conversation for the different periods under analysis were identified.

The synthesized networks and communities detected in the previous steps were further processed with Gephi software, an open-source tool of proven validity for improving the visualization and analysis of large network graphs (Bastian et al. 2009; Orbegozo-Terradillos et al. 2022). Gephi’s Force-Atlas 2 algorithm (Jacomy et al. 2014) was employed because of its usefulness in bringing nodes that form part of the same communities closer together and away from those with which they are less algorithmically related.

In the second stage of the empirical approach, artificial neural networks were used to process the tweets and achieve the goal of analyzing the sentiments generated in those tweets. Sentiment analysis, also known as opinion mining or emotion classification, is a combination of natural language processing (NLP) and text mining, with the aim of analyzing the text data of social media, among others, and having that information, mine the user’s emotions (Salloum et al. 2017; Jain and Kaushal 2018).

It has been detected that artificial neural networks totally surpass more traditional machine learning models (Jain and Kaushal 2018). Moreover, the key to achieve a high accuracy model seems to be in creating an architecture that combines different deep neural networks (Sosa 2017; Kamiş and Goularas 2019; Umer et al. 2021). Therefore, this study used a model adapted from Periwal (2021) that produces a sentiment analysis of tweets using deep learning algorithms. Thus, the architecture of the neural network model is led by the Bi-LSTM and attention as detailed below.

**Fig. 1:**
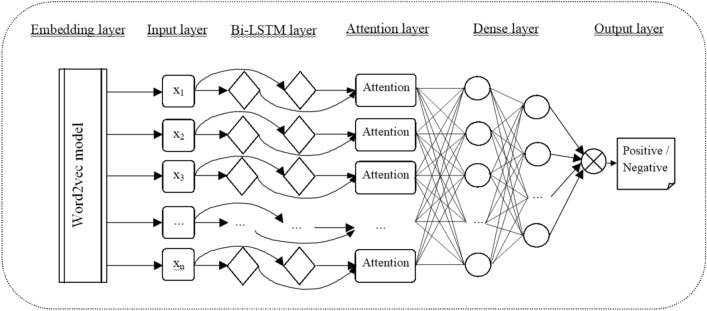
Sentiment analysis neural network model applied in the study

To do that, first of all, unprocessed text data from the sentiment140 dataset were gathered and preprocessed (Go et al. 2009; Kaggle 2022; Sentiment140 2022). This dataset contains 1,600,000 tweets extracted using the Twitter API, which have already had their sentiment classified as a positive, labeled as 1, or negative, labeled as 0. There are 800,000 tweets of each classification type respectively, which means that the dataset is not skewed. After preprocessing, data were split into a training dataset and a test dataset. Then, text was tokenized and padded. The output achieved is the input for our sentiment classification neural network model, which is a deep learning sequence model. The model has an architecture composed of the following layers: an embedding layer created using the word2vec model to convert tweets into word vectors (Mikolov et al. 2013); a Bi-LSTM neural network layer to capture the semantic meaning of the text (Elfaik and Nfaoui 2021; Chandra et al. 2021); an attention mechanism layer to extract the most relevant words; a dense layer which adds a fully connected layer in the model and that the argument passed specifies the amount of nodes in that layer; and finally, a last dense layer with a sigmoid activation function to achieve the emotion classification (Fig. 1). After training and testing the described model, it was applied to unlabeled tweets related to nuclear energy, returning the emotion of each of them. Figure 1 summarizes the used sentiment analysis neural network model (Kaggle 2022).

**Fig. 2:**
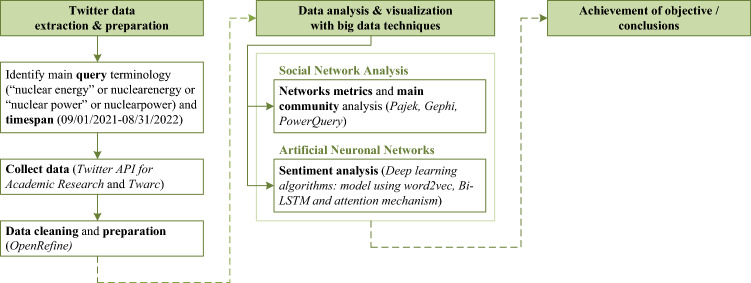
Methodological process applied in the study

Figure 2 summarizes the methodological process applied in the study.

## Results and discussion

The research collected a total sample of 3,858,024 tweets. Since English terms were used for the search, almost all the tweets were in English (96.15%), so in order to maintain homogeneity throughout the study, it was decided to work with the tweets in English, i.e., with a sample of 3,709,417 tweets.

**Fig. 3:**
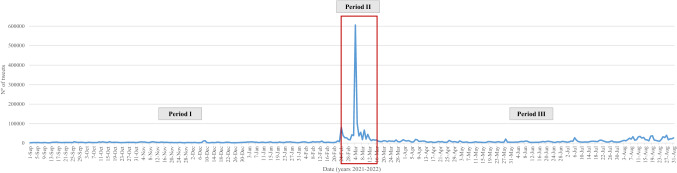
Daily trend of tweets throughout the year (from September 1, 2021, to August 31, 2022)

Figure 3 shows that, as expected, the distribution of tweets has not been homogeneous over time. Specifically, unusual activity is observed with 606,055 tweets (16.3% of total tweets) on March 4, 2022. The attack on Zaporizhzhia, Europe’s largest nuclear power plant (CNN 2022), generated global social alarm reflected in the social network activity.

Regarding the three periods under study, 18.5% of the tweets collected correspond to period I, 35.7% of the tweets correspond to period II and 45.8% of the tweets correspond to period III (Fig. 3). Thus, the impact that the conflict has had on the intensification of the digital conversation about nuclear energy can be observed.

**Fig. 4:**
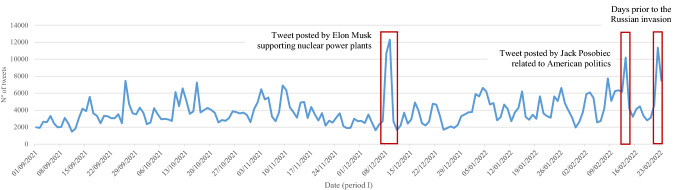
Daily trend of tweets over the first period (from September 1, 2021, to February 23, 2022)

**Fig. 5:**
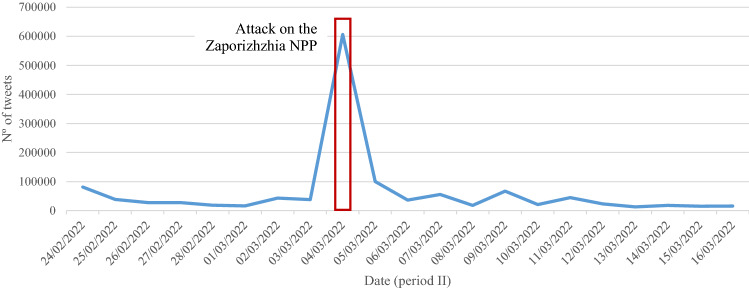
Daily trend of tweets over the second period (from February 24, 2022, to March 16, 2022)

**Fig. 6:**
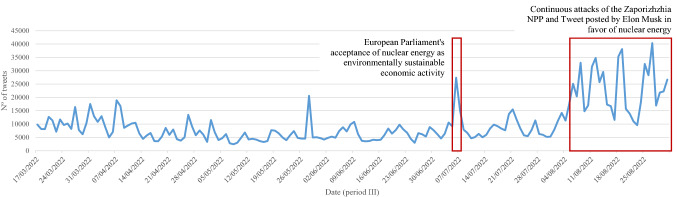
Daily trend of tweets over the third period (from March 17, 2022, to August 31, 2022)

By isolating the three periods for a clearer view of what happens in each of the periods, Figs. 4, 5 and 6 show that the distribution of tweets has not been homogeneous over time. Different protagonists, topics or events have led to increased user activity on the social network.

The first period (Fig. 4) shows odd activity on December 8 and 9, due to a tweet posted by Elon Musk supporting nuclear power plants: “*Unless susceptible to extreme natural disasters, nuclear power plants should not be shut down.*”^1^ Likewise, on February 13, an American alt-right political activist, Jack Posobiec, posted a tweet related to American politics, which had a great response: “*Biden’s National Security Advisor was involved in a illegal spying operation on the previous President. He is now escalating tensions with a nuclear power in Eurasia bc Biden’s poll numbers are down.*”^2^ In addition, on February 22, the proximity of the outbreak of the Russian invasion is noted on the network.

The second period (Fig. 5), as previously emphasized, shows the impact of the Russian invasion of Ukraine on network activity related to nuclear energy and the great social upheaval generated by the attack on the Zaporizhzhia NPP.

In the last period (Fig. 6), the unusual network activity on July 6 is due to the European Parliament's acceptance of the inclusion of nuclear energy and gas as environmentally sustainable economic activities (European Parliament 2022). Finally, the upturn in the number of tweets in August is, above all, due to the upsurge of continuous Russian attacks of the Zaporizhzhia NPP and tweets in favor of nuclear energy posted by influential actors such as Elon Musk: “*Countries should be increasing nuclear power generation! It is insane from a national security standpoint & bad for the environment to shut them down.*”^3^

All in all, it is observed that the role of influencers such as Elon Musk is crucial for spreading messages.

### Networks metrics and main community analysis (RQ-1, RQ-2, RQ-3)

#### Network metrics

Metrics extracted from the analyzed conversations show the main characteristics of the conversations about nuclear energy in the different periods and morphological differences between them (Table 1).

**Table 1:** Metrics extracted from the analyzed conversations (Zarrabeitia-Bilbao et al. 2022b)

Metrics analyzed	Overall networks for different periods			
	Global	Period I	Period II	Period III
Total impacts (tweets or retweets)	3,709,417	684,961	1,325,015	1,699,441
Users (nodes)^a^	1,243,554	350,377	574,937	668,543
Average impacts (per user)	2.98	1.95	2.3	2.54
Arcs (interactions)	3,767,699	758,625	1,436,134	1,697,983
Density	0.00000244	0.00000618	0.00000434	0.00000380
Average degree	6.05956637	4.33033561	4.99579606	5.07965232
Maximum distance	28	24	28	32
Average distance	7.27924	6.64913	10.04885	7.61276
Input degree centralization	0.07459838	0.05662457	0.07531547	0.06023185
Output degree centralization	0.00236256	0.00425212	0.00143234	0.00362949
Betweenness centralization	0.01229997	0.00685957	0.00209164	0.00790441
Number of clusters	20,985	12,186	9,326	13,388
Modularity (Louvain multi-level algorithm)	0.688192	0.760213	0.665745	0.717920

^a^Number of users who have participated in the conversation, i.e., only users who have interpellated or have been interpellated.

As mentioned in the previous section, the number of impacts in the second period (1,325,015 impacts in 3 weeks) was relatively large (35.7% of the impacts for the whole year). This was somewhat foreseeable due to the media and social commotion generated by the Russian invasion of Ukraine and the attack on the Zaporizhzhia NPP.

It can be seen that the most cohesive network is that of the first period (density 0.000618%); however, the low-density figures indicate that there are still many strategic interpellations to be explored by the nuclear energy ecosystem. Moreover, input degree centralization is higher in the case of the second period (7.53%) than for the other periods. The group of nodes that receive many mentions is larger than in the rest of the cases, placing us in a scenario of a less horizontal dialogic. However, it is observed that all centrality values are low. Thus, it is inferred, both for the different periods and for the overall set, that small groups of actors did not capitalize the reception of mentions in the digital discussions (input degree centralization); that there was no single group that posted most of the mentions (output degree centralization); and that the digital network is distributed horizontally rather than being monopolized by a few users (betweenness centralization).

Finally, in all cases, the modularity is greater than 0.3, which indicates, for the whole time span studied, a community structure of great mathematical significance (Newman and Girvan 2004).

All in all, except for the high relative activity in the second period, there are no significant morphological differences in the networks of the different time spans analyzed.

#### Main community analysis

**Fig. 7:**
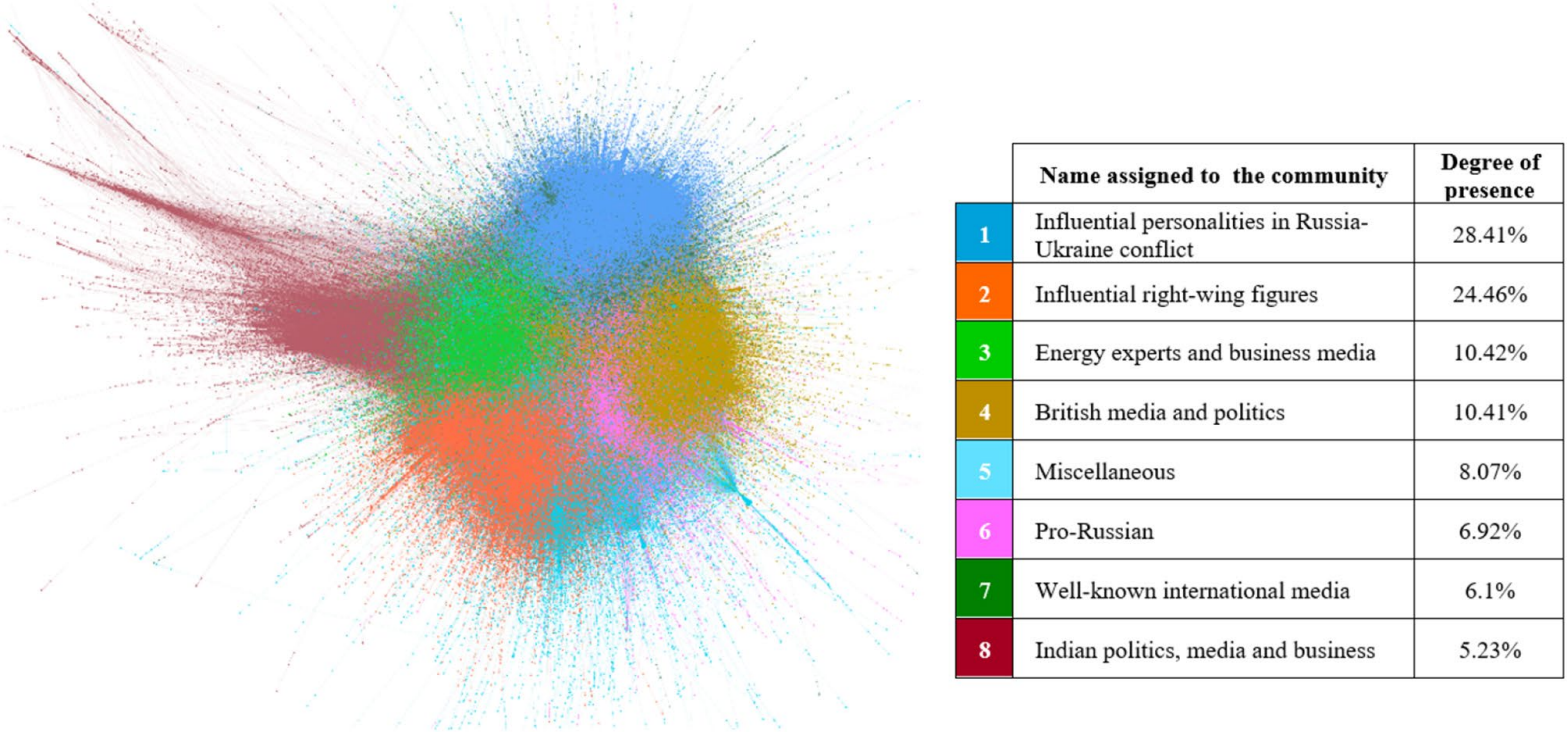
Most important communities’ network throughout the year (from September 1, 2021, to August 31, 2022)

**Fig. 8:**
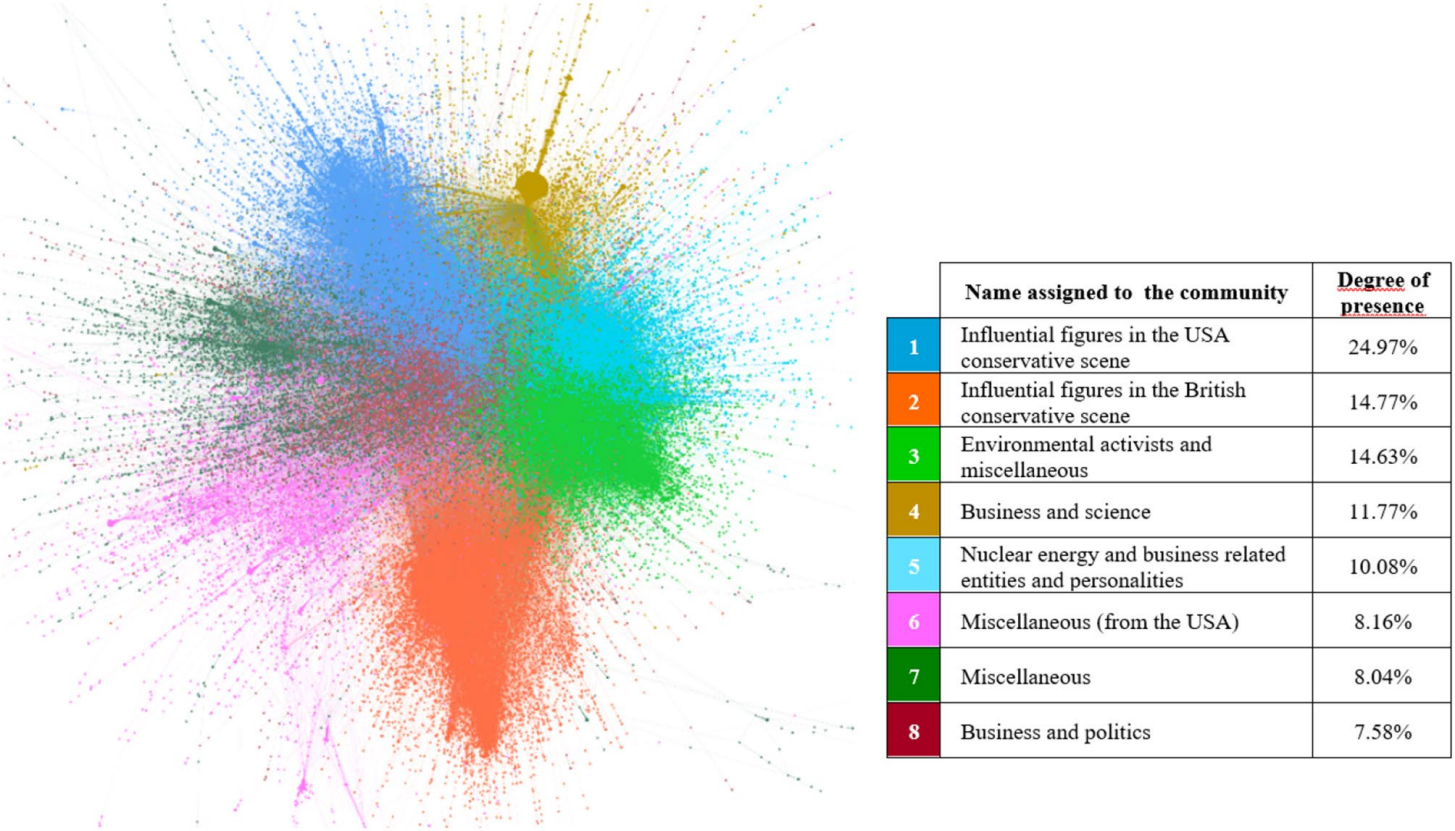
Most important communities’ network over the first period (from September 1, 2021, to February 23, 2022)

**Fig. 9:**
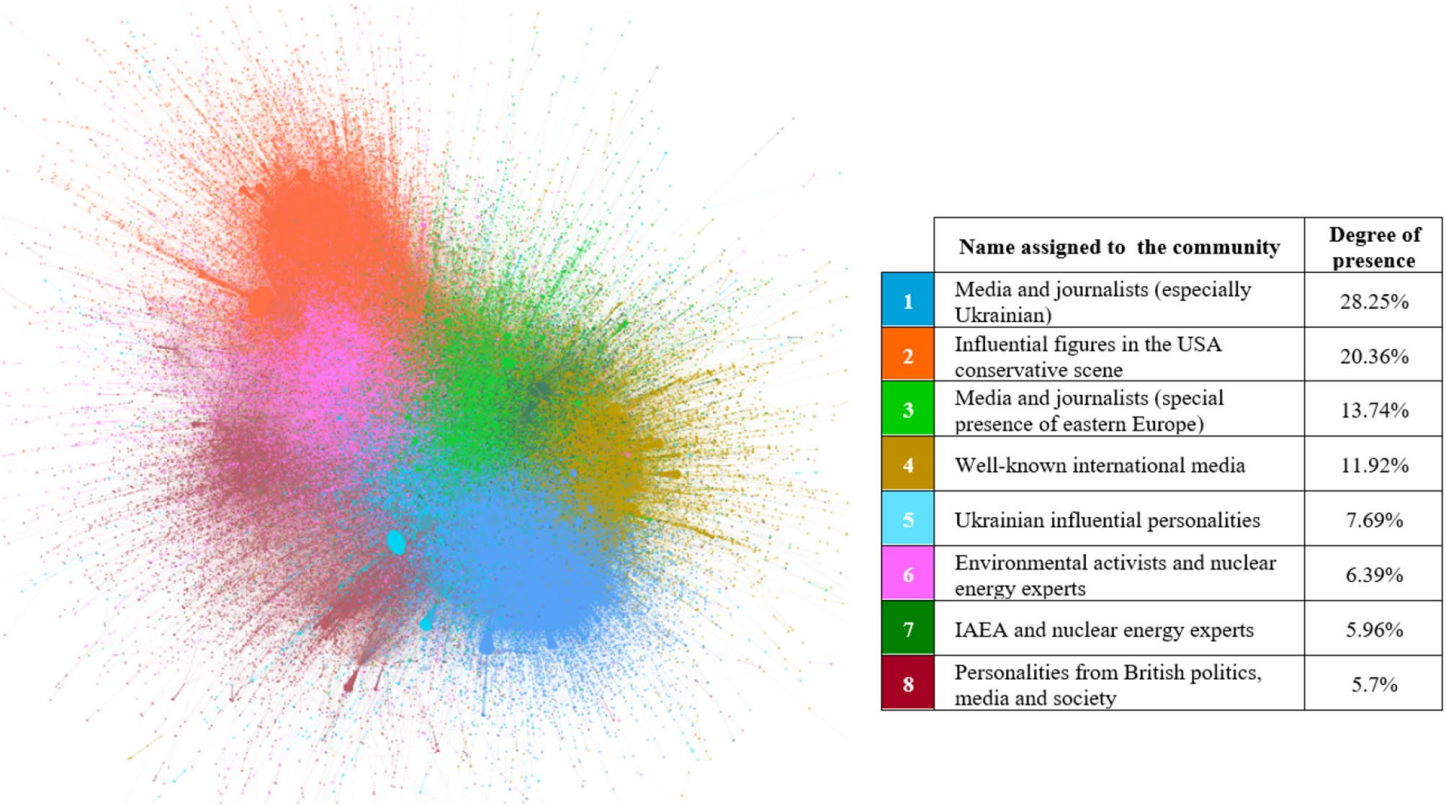
Most important communities’ network over the second period (from February 24, 2022, to March 16, 2022)

**Fig. 10:**
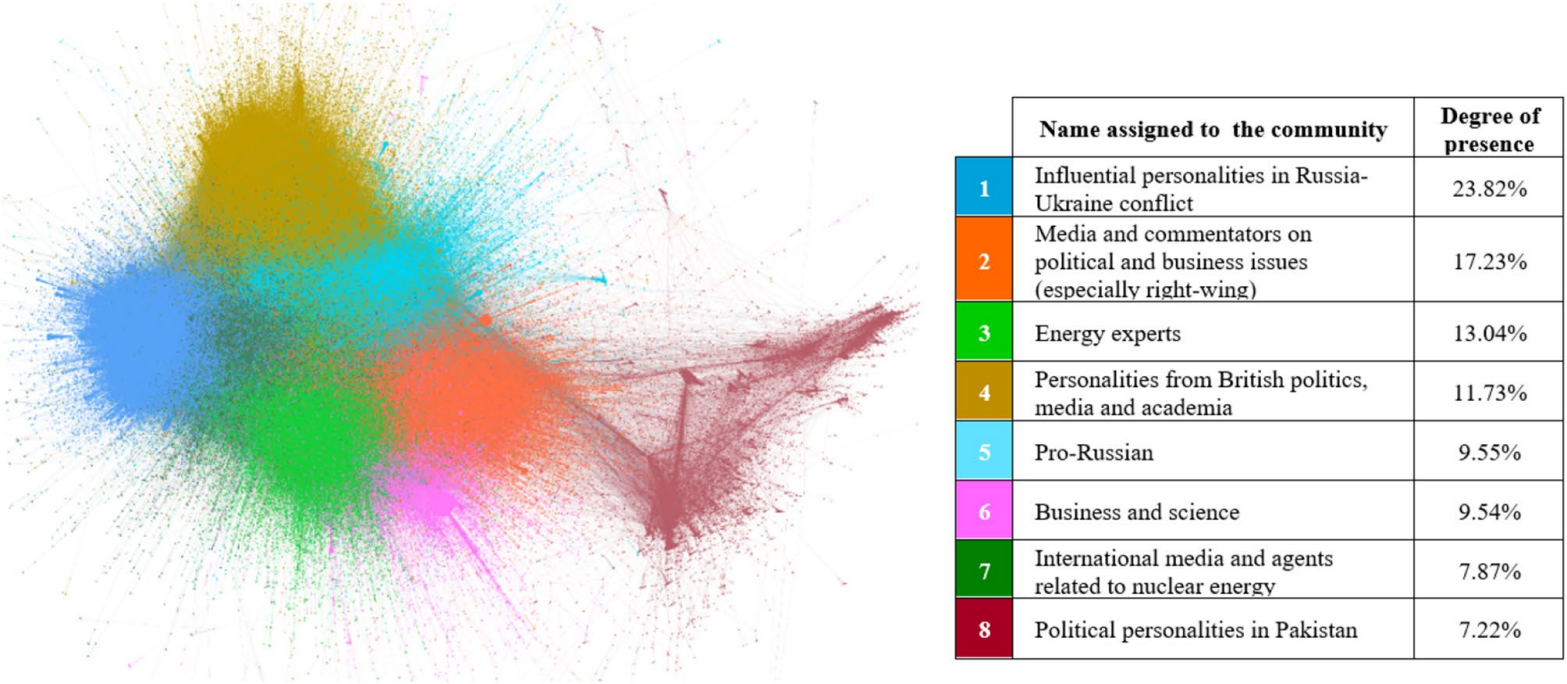
Most important communities’ network over the third period (from March 17, 2022, to August 31, 2022)

Regarding the central characters of the digital discussion, Figs. 7, 8, 9 and 10 illustrate, per different time span studied, which eight main communities (representing in all cases more than 3.4% of the actors in the network) have participated in the digital conversation and the position that each community holds.^4^

Tables 2, 3, 4 and 5 show who the key players of the conversation and what the main topics discussed in each of the communities have been. On the one hand, the analysis of the protagonists of the conversations has been conducted by analyzing both the unweighted and weighted input degree. The first of these metrics indicates the number of “neighbors” a user has without counting the number of times the user talks to the “neighbors”, i.e., which users have the largest number of unique audiences. However, this metric is combined with the weighted input degree, since for long study periods, it indicates that a user is important not only in a conjunctural way, having only once been retweeted a lot, but also in a structural way. And, on the other hand, the content that has been important in each of the communities has been determined by carefully reading the most “viralized” tweets in each community.

**Table 2:** Brief description of the main communities’ throughout the year (from September 1, 2021, to August 31, 2022)

	Leaders		Main topics/messages
	Input degree	Weighted input degree	
1	@kyivindependent (64,976) @dmytrokuleba (36,578) @iaponomarenko (26,286) @zelenskyyua (23,962) @olgatokariuk (17,268)	@kyivindependent (160,690) @dmytrokuleba (37,640) @iaponomarenko (31,401) @nexta_tv (31,107) @zelenskyyua (29,075)	Russia–Ukraine conflict. Attack on the Zaporizhzhia NPP
2	@elonmusk (92,770) @shellenbergermd (13,597) @disclosetv (13,342) @petersweden7 (12,924) @jackposobiec (12,083)	@elonmusk (107,682) @shellenbergermd (20,467) @disclosetv (16,159) @petersweden7 (15,411) @jackposobiec (14,080)	Support for nuclear energy as a national, international and environmental strategy
3	@ziontree (7,560) @business (6,450) @wsj (5,330) @briangitt (5,098) @doombergt (3,970)	@quakes99 (17,043) @ziontree (13,410) @briangitt (9,176) @business (8,787) @energybants (6,392)	Support for nuclear energy as a national, international and environmental strategy
4	@skynews (4,751) @uklabour (4,319) @kwasikwarteng (3,701) @johnredwood (3,332) @katyjayne101 (3,041)	@dorfman_p (10,095) @skynews (5,887) @kwasikwarteng (5,727) @uklabour (5,606) @johnredwood (4,227)	UK energy policy. Criticism of those who have opposed this energy source and references to the Russian invasion of Ukraine
5	@alecstapp (8,133) @milesklee (5,537) @shoe0nhead (5,473) @dsacostanza (4,435) @reviewspossum (2,239)	@alecstapp (9,884) @shoe0nhead (6,369) @milesklee (5,539) @dsacostanza (4,471) @reviewspossum (3,812)	Messages in favor of nuclear energy and against theories that have tried to vilify it
6	@rt_com (4,799) @richimedhurst (4,273) @gonzalolira1968 (3,434) @amb_ulyanov (3,386) @antonioguterres (2,796)	@rt_com (7,200) @gonzalolira1968 (4,913) @richimedhurst (4,855) @amb_ulyanov (4,713) @antonioguterres (3,491)	Pro-Russian messages
7	@reuters (17,821) @nytimes (11,406) @cnn (10,791) @washingtonpost (5,132) @time (4,791)	@reuters (22,763) @nytimes (14,777) @cnn (13,648) @washingtonpost (6,453) @abc (5,591)	Attack on the Zaporizhzhia NPP
8	@sunandavashisht (4,692) @ani (3,856) @chellaney (3,103) @abhishbanerj (2,205) @vikramchandra (2,175)	@ani (5,220) @sunandavashisht (4,707) @chellaney (3,834) @abhishbanerj (2,436) @rishibagree (2,280)	Messages in favor of Indian policies that make India a nuclear power and references to the Russian invasion of Ukraine

**Table 3:** Brief description of the main communities’ over the first period (from September 1, 2021, to February 23, 2022)

	Leaders		Main topics/messages
	Input degree	Weighted input degree	
1	@jackposobiec (9,484) @shellenbergermd (5,798) @stillgray (5,775) @dancrenshawtx (3,395) @balajis (2,191)	@jackposobiec (10,387) @shellenbergermd (8,268) @stillgray (6,290) @dancrenshawtx (3,859) @scottadamssays (2,383)	Criticism of Joe Biden’s administration. Pro-nuclear energy messages
2	@johnredwood (2,894) @danieljhannan (2,246) @tomhfh (1,714) @kwasikwarteng (1,523) @gbnews (1,118)	@johnredwood (3,589) @danieljhannan (2,268) @kwasikwarteng (2,104) @tomhfh (2,057) @gbnews (1,309)	UK energy policy. Criticism of those who have opposed this energy source
3	@isabelleboemeke (2,333) @ziontree (2,237) @eu_commission (2,032) @greenpeace (1,675) @mboudry (1,305)	@ziontree (4,217) @isabelleboemeke (3,406) @eu_commission (2,803) @w_nuclear_news (2,492) @greenpeace (2,348)	Messages in favor of nuclear energy to face the energy crisis
4	@elonmusk (19,842) @slashdot (448) @rainmaker1973 (405) @ultrasafenuke (253) @wingod (154)	@elonmusk (20,862) @slashdot (481) @rainmaker1973 (420) @wingod (265) @ultrasafenuke (262)	Messages in favor of nuclear power plants and nuclear technology
5	@iaeaorg (2,550) @secgranholm (1,914) @business (1,830) @quakes99 (1,624) @zerohedge (1,378)	@quakes99 (,7076) @iaeaorg (5,058) @secgranholm (2,690) @business (2,315) @uraniumtrends (2,102)	Messages in favor of nuclear energy as green energy and job creator
6	@nukestrat (1,091) @benjaminnorton (979) @proustmalone (657) @sovietvisuals (629) @jimmy_dore (596)	@nukestrat (1,096) @benjaminnorton (1,037) @khamenei_ir (670) @proustmalone (657) @rt_com (643)	Russian–Ukrainian war. Criticism of American foreign policy
7	@shoe0nhead (3,819) @vers_lalune (1,019) @lilithlovett (623) @disclosetv (600) @alex_avoigt (491)	@shoe0nhead (4,369) @vers_lalune (1,030) @disclosetv (717) @lilithlovett (665) @alex_avoigt (640)	Messages in favor of nuclear energy and against theories that have tried to vilify it
8	@alecstapp (2,369) @patrickc (1,167) @davidfrum (1,144) @owasow (1,049) @elidourado (722)	@alecstapp (2,717) @davidfrum (1,355) @patrickc (1,245) @owasow (1,240) @elidourado (750)	Progress of nuclear energy and nuclear power plants

**Table 4:** Brief description of the main communities’ over the second period (from February 24, 2022, to March 16, 2022)

	Leaders		Main topics/messages
	Input degree	Weighted input degree	
1	@kyivindependent (43,304) @iaponomarenko (18,695) @olgatokariuk (13,759) @thor_benson (13,497) @phildstewart (10,993)	@kyivindependent (66,093) @iaponomarenko (20,414) @olgatokariuk (16,387) @thor_benson (14,273) @phildstewart (12,318)	Attack on the Zaporizhzhia NPP
2	@elonmusk (41,970) @cernovich (10,316) @greg_price11 (5,907) @pmarca (4,914) @shellenbergermd (3,232)	@elonmusk (43,886) @cernovich (12,499) @greg_price11 (5,939) @pmarca (5,095) @shellenbergermd (4,033)	Support for nuclear power plants as a national and international political strategy
3	@spectatorindex (14,905) @nexta_tv (13,222) @bnonews (9,220) @hannaliubakova (3,851) @conflicts (3,717)	@spectatorindex (22,972) @nexta_tv (20,859) @bnonews (13,530) @visegrad24 (4,355) @hannaliubakova (4,065)	Attack on the Zaporizhzhia NPP
4	@reuters (12,957) @afp (9,731) @cnn (8,228) @time (4,060) @cbsnews (3,831)	@reuters (15,549) @afp (11,628) @cnn (9,478) @cbsnews(4,578) @time (4,289)	Attack on the Zaporizhzhia NPP. And a tsunami warning by Japanese officials in Fukushima prefecture and wildfire broke out near a nuclear power plant in South Korea
5	@dmytrokuleba (36,334) @klitschko (8,545) @melaniepodolyak (808) @loveon999 (597) @goncharenkoua (560)	@dmytrokuleba (37,352) @klitschko (8,558) @melaniepodolyak (810) @goncharenkoua (710) @loveon999 (631)	Attack on the Zaporizhzhia NPP
6	@ziontree (3,175) @calroo1 (2,650) @jrmygrdn (1,771) @jmichaelwaller (1,379) @georgemonbiot (1,209)	@ziontree (3,810) @calroo1 (2,779) @jrmygrdn (2,091) @quakes99 (1,676) @jmichaelwaller (1,448)	Relationship between Gazprom (Russian majority state-owned multinational energy corporation) and environmental NGOs
7	@iaeaorg (30,059) @rafaelmgrossi (10,810) @iaeaiec (2,117) @james_acton32 (1,006) @innasovsun (834)	@iaeaorg (47,747) @rafaelmgrossi (150,49) @iaeaiec (2,171) @james_acton32 (1,103) @bulletinatomic (955)	Information on radiation levels at the Zaporizhzhia NPP site and status of the nuclear power plant after the attack
8	@katyjayne101 (3,038) @uklabour (2,631) @adamboultontabb (1,977) @paulembery (1,302) @lloydgb1962 (761)	@katyjayne101 (3,039) @uklabour (2,835) @adamboultontabb (1,984) @paulembery (1,312) @lloydgb1962 (817)	UK energy policy. Criticism of those who have opposed this energy source and references to the Russian invasion of Ukraine

**Table 5:** Brief description of the main communities’ over the third period (from March 17, 2022, to August 31, 2022)

	Leaders		Main topics/messages
	Input degree	Weighted Input degree	
1	@kyivindependent (32,936) @olenahalushka (10,565) @samramani2 (10,427) @zelenskyyua (9,238) @iaponomarenko (9,205)	@kyivindependent (94,452) @olenahalushka (19,327) @mhmck (17,039) @gerashchenko_en (13,471) @samramani2 (12,741)	Continuous Russian attacks of the Zaporizhzhia NPP
2	@disclosetv (12,625) @petersweden7 (12,170) @shellenbergermd (6,337) @zerohedge (5,043) @wallstreetsilv (4,001)	@disclosetv (15,228) @petersweden7 (14,372) @shellenbergermd (8,166) @zerohedge (6,355) @wallstreetsilv (4,519)	Messages in favor of nuclear energy as green energy and criticism of policies that do not support nuclear energy
3	@briangitt (4,270) @doombergt (3,717) @ziontree (3,222) @energybants (2,972) @sstapczynski (2,904)	@quakes99 (8,291) @briangitt (7,742) @ziontree (5,383) @doombergt (5,254) @energybants (4,661)	Support for nuclear energy as a national and environmental strategy
4	@borisjohnson (6,066) @skynews (2,408) @kwasikwarteng (2,069) @pickardje (1,806) @premnsikka (1,764)	@borisjohnson (7,270) @kwasikwarteng (3,043) @skynews (2,869) @uklabour (2,437) @msm_monitor (1,967)	General policy and energy policy in particular, in favor of nuclear energy
5	@gonzalolira1968 (3,434) @richimedhurst (3,390) @amb_ulyanov (3,088) @levi_godman (2,272) @rt_com (2,129)	@gonzalolira1968 (4,913) @amb_ulyanov (4,316) @richimedhurst (3,641) @levi_godman (3,316) @rt_com (3,205)	Pro-Russian messages
6	@elonmusk (40,270) @engineers_feed (1,271) @rainmaker1973 (909) @stats_feed (443) @ppathole (361)	@elonmusk (42,934) @engineers_feed (1,355) @rainmaker1973 (949) @stats_feed (455) @worldandscience (449)	Support for nuclear energy as a national, international and environmental strategy
7	@iaeaorg (1,4513) @nytimes (5,265) @reuters (4,290) @rafaelmgrossi (4,278) @ap (3,103)	@iaeaorg (24,923) @nytimes (7,515) @rafaelmgrossi (7,050) @reuters (5,630) @un (4,331)	Russia–Ukraine conflict. Continuous Russian attacks of the Zaporizhzhia NPP
8	@naya__pakistan_ (4,904) @cmshehbaz (2,526) @arifcrafiq (2,088) @imrankhanpti (1,848) @hammad_azhar (1,507)	@naya__pakistan_ (4,904) @cmshehbaz (2,554) @arifcrafiq (2,089) @imrankhanpti (1,931) @hammad_azhar (1,512)	Messages in favor of Pakistani policies that make Pakistan a nuclear power

First of all, it can be seen that there is alignment between the agents with the highest input degree and the highest weighted input degree. In addition, no account displays, a priori, suspicious behavior. No user has a suspiciously high weighted input degree level compared with the unweighted input degree level, so that, a priori, no account is artificially promoted by other automated accounts.

From the community and leader analysis carried out, it can be inferred that, in general, actors related to politics, business, science and media lead the digital discussions about nuclear energy on Twitter. Therefore, this social network is a dissemination tool that works, in this case, as a means of expression for specific communities: Namely technical or collegiate communities, with a knowledge or interest in the topic, and that support nuclear energy as a national, international and environmental strategy.

In addition, influencers who generate unusual activity on the network (Figs. 3 and 5), as might be expected, also attract a great deal of interactivity. Specifically, it should be noted that Elon Musk is the predominant profile in the digital conversation as a whole; expressly, he ranks first among all actors in terms of unweighted input degree and second in terms of weighted input degree (Table 2).

If the evolution of the different periods is analyzed, as expected, the Russian invasion of Ukraine and the attack on the Zaporizhzhia NPP has changed the communities and leaders that are part of the digital discussions. Being an armed conflict, from the second period onward, the stakeholders involved in the conflict appear (stakeholders related to Ukraine and Russia) and the media gains a strong presence, i.e., nuclear energy and nuclear power plants become news. Nuclear energy loses interest as a technology and gains interest as a current event.

An absence of nuclear energy rejection groups is observed in the digital sphere. It can be said that the debate about nuclear energy is, in fact, practically a taboo within the environmental movements; and a great niche for conservatives.

Moreover, it seems that the borders between countries (the USA, UK, India, Pakistan) to a certain degree also mark the relationships within the network. However, in general, all communities are interconnected, i.e., the networks are not polarized.

### Sentiment analysis (RQ-4)

After crossing each layer of the neural networks, the sentiment model returns the emotion of the social media text data, detecting whether each contained tweet shows a positive or a negative sentiment. The presented model gave an accuracy of 84.97%, surpassing other existing models for this purpose (Rustam et al. 2019; Albaldawi and Almuttairi 2020).

**Fig. 11:**
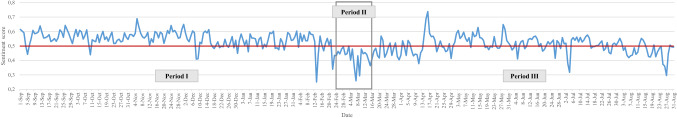
Average tweet sentiment per day throughout the year (from September 1, 2021, to August 31, 2022)

**Fig. 12:**
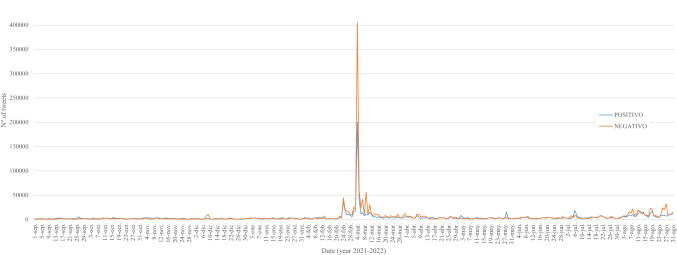
Number of positive and negative tweets per day throughout the year (from September 1, 2021, to August 31, 2022)

**Fig. 13:**
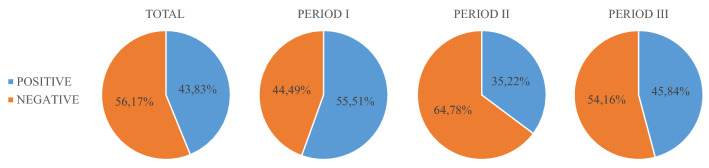
Ratio of positive and negative tweets through different periods

Thus, on the one hand, Fig. 11 shows the average sentiment (from 0, negative, to 1, positive) of tweets per day. On the other hand, Fig. 12 shows the number of positive and negative tweets (sentiment score higher or lower than 0.5) per day and Fig. 13 shows the ratio of positive (sentiment score higher than 0.5) and negative (sentiment score lower than 0.5) tweets for the different time span analyzed.

Previous studies show that the debate in the digital sphere is positive when talking about green energy (Zarrabeitia-Bilbao et al. 2022a); however, in this case, it is observed that this is not the general tendency. In the first period, although there were negative peaks, such as the one on February 13 (Fig. 11), mainly due to the viralization of the previously mentioned tweet sent by Jack Posobiec (see footnote 2), the tendency is positive, but the emotional trend changes drastically with the beginning of the Russian invasion of Ukraine. As expected in a war setting, the messages become negative, and even more so in this case, when the topic of conversation revolves around an attack on a nuclear power plant and the energy problems resulting from dependence on Russian gas. In the third period, the negative sentiment is less pronounced; however, the most positive peak, on April 16 (Fig. 11), is mainly due to a tweet praising wind energy to combat climate change^5^ and the most negative peaks continue to be related to the Russian invasion and the ongoing attacks of the Zaporizhzhia NPP.

## Conclusions, limitations and future works

Governments, businesses and society are joining forces in climate initiatives to accelerate climate action (SDG goal 13). In this sense, energy transition is the most decisive driver of change to reverse climate change. However, the energy transition faces several setbacks. The global pandemic caused by COVID-19 and Russia’s invasion of Ukraine have had a huge impact on different markets and, in particular, on energy markets. In this scenario, one source of energy that has been talked about a lot in recent times is nuclear energy. A source of energy or a topic of conversation has not been without controversy in recent years.

In a topic of this magnitude, understanding the impact and the social trend that is taking place is crucial. In this sense, the microblogging platform Twitter acts as a social thermometer and can reflect the trends, opinions or concerns of society, in general, and more specific, technical and controversial ecosystems such as nuclear energy, in particular.

In this case, as is logical, the digital conversation around nuclear energy is influenced by events of a “macro” nature, such as the Russian invasion of Ukraine, which has global consequences, especially in the field of international relations and global energy supply. However, a significant fact is that simple tweets containing personal and political opinions of influential people also manage to intervene in the digital conversation defining the magnitude and direction of the debate. This shows the volatility of the conversation analyzed.

Moreover, it can be said that the digital conversation around nuclear power is not constructed as a public controversy. The data show that it is a conversation, in general, with non-polarized actors. The interactions analyzed and the communities derived do not describe a conversation around communities at odds with each other, with disparate and confrontational opinions. In fact, there is more of a strategic alliance between those communities that discuss the topic in scientific, technological, political and business terms. This alliance is not even questioned with the outbreak of the Russian invasion and the attack on the Zaporizhzhia NPP; indeed, neither armed conflict nor military threats against nuclear power plant succeed in stirring up anti-nuclear voices, even though these events do modify the orientation of the sentiment in the language used, making it more negative.

As a limitation of the study, it should be noted that the social network Twitter is also associated with a tendency toward digital endogamy and the creation of digital niches, which the scientific community refers to as echo chambers (González 2011). Moreover, other limitations shown by Twitter are due to the bias of the data collected, the representation bias when making general assumptions and the language employed by users. Nevertheless, despite the inherent limitations of any study, in this case, through the unique research perspective that Twitter offers to the scientific community, it has been possible to address the objective of social listening analysis of nuclear energy.

Finally, by way of future works related to this research, it would be interesting to further explore the discussions held in the different communities through content analysis techniques; using other deep learning techniques to analyze the different emotions reflected in conversations and their intensity; and extend the research to identify the development trends in science and technology, through scientometric and patentometric techniques, using data from scientific and patent databases.

## Footnotes

1 https://twitter.com/elonmusk/status/1468689628161052674.

2 https://twitter.com/jackposobiec/status/1492874434725744643.

3 https://twitter.com/elonmusk/status/1563292201043431424.

4 The order of the colors will be maintained for all the networks and will be in accordance with the size of the community (from largest to smallest). Therefore, the fact that a color is repeated from one network to another does not mean that it is the same community, in fact, from one network to another there will always be differences in the communities.

5 https://twitter.com/occupydemocrats/status/1515057469944320000?lang=bg.
